# Novel testing strategy for prediction of rat biliary excretion of intravenously administered estradiol-17β glucuronide

**DOI:** 10.1007/s00204-020-02908-x

**Published:** 2020-11-07

**Authors:** Annelies Noorlander, Eric Fabian, Bennard van Ravenzwaay, Ivonne M. C. M. Rietjens

**Affiliations:** 1 Division of Toxicology, Wageningen University and Research, Stippeneng 4, 6708 WE Wageningen, The Netherlands; 2 Experimental Toxicology and Ecology, BASF SE, Ludwigshafen, Germany

**Keywords:** Physiologically based kinetic modelling, Biliary excretion, Primary rat hepatocytes, Scaling factor

## Abstract

The aim of the present study was to develop a generic rat physiologically based kinetic (PBK) model that includes a novel testing strategy where active biliary excretion is incorporated using estradiol-17β glucuronide (E_2_17βG) as the model substance. A major challenge was the definition of the scaling factor for the in vitro to in vivo conversion of the PBK-model parameter *V*_max_. In vitro values for the *V*_max_ and *K*_m_ for transport of E_2_17βG were found in the literature in four different studies based on experiments with primary rat hepatocytes. The required scaling factor was defined based on fitting the PBK model-based predicted values to reported experimental data on E_2_17βG blood levels and cumulative biliary E_2_17βG excretion. This resulted in a scaling factor of 129 mg protein/g liver. With this scaling factor the PBK model predicted the in vivo data for blood and cumulative biliary E_2_17βG levels with on average of less than 1.8-fold deviation. The study provides a proof of principle on how biliary excretion can be included in a generic PBK model using primary hepatocytes to define the kinetic parameters that describe the biliary excretion.

**Electronic supplementary material:**

The online version of this article (10.1007/s00204-020-02908-x) contains supplementary material, which is available to authorized users.

## Introduction

The kinetic profile of a substance is of importance when considering human safety assessment and drug development. Prediction of these kinetic profiles based on absorption, distribution, metabolism and excretion (ADME) using physiologically based kinetic (PBK) modelling has been shown of value not only to predict blood levels of drugs following defined dose levels in forward dosimetry, but also in so-called reverse dosimetry for quantitative in vitro—in vivo extrapolation (QIVIVE) (Louisse et al. 2017; Rietjens et al. 2011). QIVIVE aims to contribute to the 3Rs of Russel and Burch (reduction, replacement and refinement) of animal experiments (Flecknell 2002; Louisse et al. 2015; Zhang et al. 2018). Up to now, proofs of principle for human safety assessment using PBK model-facilitated QIVIVE have been mainly provided for chemicals that do not depend on active excretion via either kidneys or liver (Fabian et al. 2019; Louisse et al. 2010; Moxon et al. 2020; Ning et al. 2019; Punt et al. 2019; Strikwold et al. 2017).

In drug development, there already have been studies on QIVIVE using PBK-modelling focussing on especially excretion of certain drugs via active transport from the liver to the bile (Chapy et al. 2015; Jamei et al. 2014; Jones et al. 2012). In addition, both endogenous and exogenous glucuronide conjugates have also been shown to be actively excreted via this route (Cronholm et al. 1971; Ge et al. 2016; Hjelle and Klaassen 1984). The common properties of the compounds for which this active transport from liver into bile was shown important are that they have a low membrane permeability, a molecular weight cut off of 475 Da (400 Da for rats), remain mostly unchanged and therefore are excreted via active uptake and efflux in and from the liver cells into bile (Yang et al. 2009).

Given these structural characteristics it can be foreseen that biliary excretion may not only be relevant for drugs but also for other chemicals, including new chemicals (Choi et al. 2019). For these substances, active excretion should be included in the PBK-models to obtain an adequate description of their kinetics and subsequent prediction of in vivo effective dose levels. However, due to the lack of well-established and validated in vitro assays to quantify kinetics for active excretion there is a lack of PBK-models including active biliary and/or renal excretion. When not including active excretion in QIVIVE, the prediction of the time-dependent blood concentration can deviate from the in vivo situation resulting in incorrect determination of points of departure for human safety assessment when based on PBK model-facilitated reverse dosimetry (Louisse et al. 2017).

**Fig. 1:**
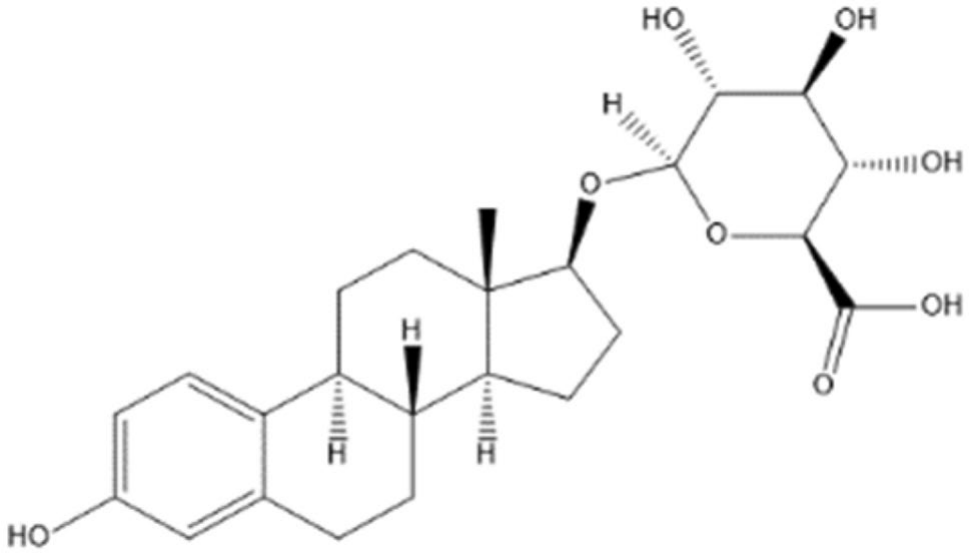
Chemical structure of estradiol-17β glucuronide

To be of value for novel non-animal based testing strategies, parameters required for the PBK-models should preferably be defined using in silico and in vitro approaches. When using in vitro models for biliary excretion, important challenges relate to the type of in vitro model to be used and the subsequent translation of the in vitro data to the in vivo situation using adequate scaling factors (Choi et al. 2019). When using for example a cell model with overexpression of a transporter of interest, proper scaling depends on the expression level of the transporter in the cell model compared to its expression level in the organ in vivo. To solve this matter a relative expression factor (REF) can be used (Chan et al. 2019; Jamei et al. 2014). Moreover, because the activity of the transporter in the transfected cell can differ from its in vivo activity often a relative activity factor (RAF) is used as well (Izumi et al. 2018; Poirier et al. 2009). Other factors may further complicate this in vitro to in vivo scaling, such as the fact that the cell of origin used to generate the transfected cell model [e.g. human embryonic kidney cells, Chinese hamster ovaries, *Xenopus Laevis* oocytes, (Cattori et al. 2001; Eckhardt et al. 1999; van de Steeg et al. 2013)] may not fully represent the cell type of the designated organ making the in vitro to in vivo translation more complex. This implies that hepatocytes may provide an alternative model to define the kinetic parameters for biliary excretion (Cantrill and Houston 2017; Chu et al. 2013; Yabe et al. 2011). Therefore, the aim of the present study was to develop a generic rat PBK model incorporating a novel testing strategy of including active biliary excretion, focussing on hepatocytes as the in vitro cell model *and* the scaling factor to be used to obtain adequate in vitro to in vivo translation. This was done using estradiol-17β glucuronide (E_2_17βG) (Fig. 1) as the model substance (molecular weight 448.5 g/mole). E_2_17βG is an anionic endogenous oestrogen glucuronide conjugate well known for its high affinity for active transport in the liver where it is taken up from the blood (sinusoidal side) via the (rat) organic anion transporting polypeptides (Oatps) into the hepatocytes, to be subsequently excreted into the bile (Kanai et al. 1996). This uptake of E_2_17βG into the hepatocytes via the Oatps is the rate-limiting step for its elimination (Varma et al. 2012).

## Materials and methods

### Physiologically based kinetic (PBK) modelling

**Fig. 2:**
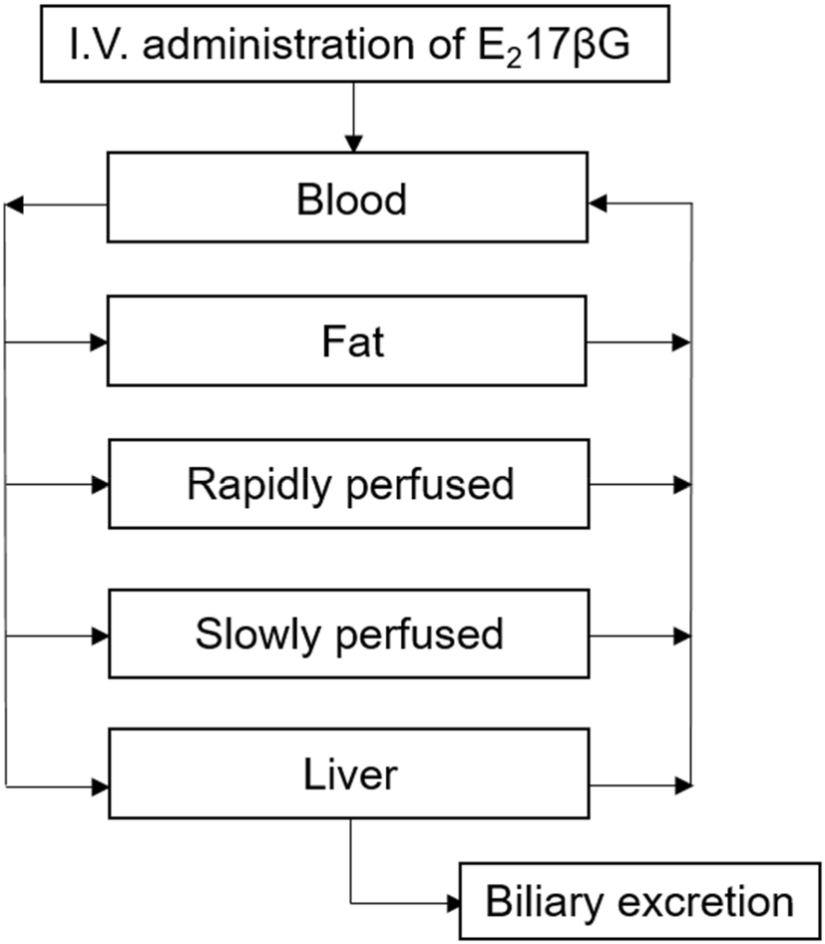
Schematic overview of the PBK model for E_2_17βG including biliary excretion

A conceptual PBK model was developed for E_2_17βG in rat (Fig. 2). The model contained separate compartments for blood, fat and liver. All other organ tissues were divided in either a rapidly perfused tissue compartment (brain, heart, lungs, kidneys) or a slowly perfused tissue compartment (bone, skin, muscle). The gastro-intestinal tract was not included as a separate compartment since administration of E_2_17βG was intravenously. Physiological and anatomical parameters such as tissue blood flow and tissue weight were obtained from Brown et al. (1997). Tissue:blood partition coefficients were determined by a mathematical method described by DeJongh et al. (1997) based on the log Kow of E_2_17βG (2.05 ALOGPS). Table 1 presents a detailed overview of the parameters.

**Table 1:** Physiological and physico-chemical parameters used in the model code

Physiological and physico-chemical parameters	Rat
Body weight (kg)	0.250
Tissue volume fraction of the body weight^a^	
Fat tissue	0.07
Liver tissue	0.034
Fraction of the blood	0.074
Rapidly perfused tissue	0.098
Slowly perfused tissue	0.724
Cardiac output (L/h)	15 × BW^0.74^
Blood flow fraction^a^	
Fat	0.07
Liver	0.174
Rapidly perfused tissues	0.234
Slowly perfused tissues	0.522
Tissue:blood partition coefficients^b^	
Fat:blood	19.87
Liver:blood	1.33
Rapidly perfused tissue:blood	1.33
Slowly perfused tissue:blood	0.60

^a^Brown et al. (1997)

^b^DeJongh et al. (1997)

The transport of E_2_17βG from liver to bile was described by Michaelis–Menten kinetics using the following formula:

1 $$\mathrm{AL}=\mathrm{QL}\times \left(\mathrm{CB}-\mathrm{CVL}\right)-\left({{V}_{\mathrm{max}}}_{{\mathrm{E}}_{2}17\mathrm{bG}} \times \frac{\mathrm{CVL}}{\left({{K}_{\mathrm{m}}}_{{\mathrm{E}}_{2}17\mathrm{bG}}+\mathrm{CVL}\right)}\right),$$

where AL is the change in the amount of E_2_17βG in the liver over time (µmol/h), QL the blood flow to the liver (L/h), CB the concentration of E_2_17βG in arterial blood (µmol/L), CVL the venous concentration of E_2_17βG in the liver (µmol/L), *V*_maxE217bG_ and *K*_mE217bG_ the maximum rate (µmol/h) and Michaelis–Menten constant (µmol/L) for the transport of E_2_17βG. The model equations were encoded and solved using Berkeley Madonna 8.3.18 (UC Berkeley, CA, USA).

Although it is known that E_2_17βG undergoes enterohepatic circulation, this was not taken into account in the model because for all the in vivo data included in this study the bile duct of the rats was cannulated, therefore, enterohepatic circulation could not take place.

### In vitro kinetic data

The in vitro kinetic data for transport of E_2_17βG into hepatocytes were obtained from the literature. Four studies, (Brock and Vore 1984; Brouwer et al. 1987; Ishizuka et al. 1998; Kouzuki et al. 1999) reported *V*_max_ (pmol/min/mg protein) and *K*_m_ (µM) values for the transport of E_2_17βG into hepatocytes. Briefly, all studies used freshly isolated primary hepatocytes from Sprague–Dawley rats and radio-labelled E_2_17βG. Three studies used a hepatocyte suspension system where concentration dependent uptake of [^3^H]-E_2_17βG under linear conditions with respect to time was determined. At the end of each incubation, a fraction of hepatocyte suspension was removed from the incubations. This fraction was added to a tube containing silicon oil and 3 M KOH. The tube was centrifuged and cut at the silicone oil layer. The hepatocyte pellet was placed in a scintillation vial and radioactivity was measured. The other study cultured the freshly isolated hepatocytes on collagen-coated dishes. After washing the dishes three times with either a Krebs–Henseleit buffer or choline buffer, the uptake was initiated by adding [^3^H]-E_2_17βG. The uptake was stopped and cells were washed three times using ice-cold Krebs–Henseleit buffer for both procedures. The cells were solubilised in 1 N NaOH, distilled water was added and [^3^H]-E_2_17βG was measured with liquid scintillation. Protein content in all studies was determined using either the method of Lowry (Lowry et al. 1951) or the method of Bradford (Bradford 1976) with bovine serum albumin as a standard. The *V*_max_ and *K*_m_ values were determined by plotting the rate of uptake (pmol/min/mg protein) of [^3^H]-E_2_17βG by the hepatocytes against the [^3^H]-E_2_17βG concentration (µM) fitting the curve using the Michaelis–Menten equation.

### Scaling factor

In the present study the scaling factor was determined by fitting the PBK model-based predicted values for blood concentrations and the cumulative biliary excretion of E_2_17βG to the data reported for these endpoints in rat experiments found in literature. Table 2 presents an overview of available in vivo studies. They included in vivo rat kinetic data for the time-dependent plasma concentration of E_2_17βG upon an intravenous dose level of 81 ng/kg bw (Gotoh et al. 2002; Morikawa et al. 2000) and 23 ng/kg bw (Slikker et al. 1983) and the time-dependent cumulative biliary excretion of E_2_17βG upon an intravenous dose level 81 ng/kg bw (Gotoh et al. 2002; Morikawa et al. 2000) and 48 ng/kg bw (Takikawa et al. 1996). Three of these studies used male Sprague–Dawley rats, one study used female Sprague–Dawley rats (Slikker et al. 1983). All studies quantified [^3^H]-E_2_17βG by liquid scintillation counting. Since the PBK model predicts whole blood concentrations and the in vivo data present time-dependent plasma concentrations, the plasma concentrations were converted to whole blood concentrations using the following formula:

2 $${C}_{\mathrm{blood}}={C}_{\mathrm{plasma}}\times \left(1-Hct\right),$$

where *C*_blood_ is the concentration of E_2_17βG in whole blood (µmol/L), *C*_plasma_ the concentration of E_2_17βG in plasma (µmol/L) and Hct the rat haematocrit, which was set at 40%, the average of the range published (Probst et al. 2006). (See figure S1 in supplementary material A for original plasma concentration data).

**Table 2:** Overview of in vivo studies reporting time-dependent blood concentration and cumulative biliary excretion upon intravenously administered E_2_17βG in rats

Dose (ng/kg bw)	Time of sample collection (h)	References
Time-dependent plasma concentration		
81	0–1	Morikawa et al. (2000)
81	0–1	Gotoh et al. (2002)
23	0–1.5	Slikker et al. (1983)
Cumulative biliary excretion		
81	0–2	Morikawa et al. (2000)
81	0–2	Gotoh et al. (2002)
48	0–1.5	Takikawa et al. (1996)

Comparison of the time-dependent plasma concentration reported by Morikawa et al. (2000) and Gotoh et al. (2002) using a similar dose of 81 ng/kg bw, indicated the data point at *t* = 1 h reported by Gotoh et al. (2002) to be an outlier (figure S1 supplementary material A), and this data point was aligned with that from Morikawa et al. (2000) before further use of the Gotoh et al. (2002) data. In addition, because the time-dependent plasma concentrations reported by Slikker et al. (1983) at a dose of 23 ng/kg bw completely overlapped with both in vivo data sets at a dose of 81 ng/kg bw (figure S2 supplementary material A), these data were corrected to bring them in line with the rest of the data by correcting the blood concentrations by a factor of 0.284 (23/81).

Combining the four in vitro kinetic data sets for the *V*_max_ and *K*_m_ of E_2_17βG transport in rat hepatocytes with the six in vivo data sets on plasma E_2_17βG and on cumulative biliary excretion of E_2_17βG provided 24 fitted scaling factors, which were combined to generate a mean value for further PBK model-based predictions. All predicted curves and reported in vivo data were graphically illustrated using GraphPad Prism 5 (GraphPad Software Inc., San Diego, CA, USA).

To define the scaling factor as described above in vitro *V*max values expressed in pmol/min/mg protein have to be converted to an in vivo *V*_max_ value expressed in µmol/h/whole liver. This was done using the following formula:

3 $$\mathrm{In vivo }{V}_{\mathrm{max}}=\left(\frac{\mathrm{In vitro }{V}_{\mathrm{max}}}{\mathrm{1,000,000}}\right)\times 60\times \mathrm{SF}\times \mathrm{Volume of liver}\times 1000,$$

where the factor 1,000,000 is used to convert pmol to µmol, 60 to convert minutes to hours, SF is the scaling factor expressed in mg protein/g liver, multiplied by the volume of the liver expressed in kilograms (Table 1), which is multiplied by 1000 to convert kilograms to grams.

The full model code is presented in Supplementary material B.

### Sensitivity analysis

To assess the influence of the scaling factor on the model predictions and to assess the model parameters that can influence the model output most, a sensitivity analysis was performed for both the predicted concentration of E_2_17βG in blood and its predicted cumulative biliary excretion. To carry out the sensitivity analysis, a dose level of one of the available rat studies was used, 81 ng/kg bw. The analysis was performed using the values for *K*_m_ and *V*_max_ obtained from the rat hepatocyte study with the lowest catalytic efficiency (CE) (calculated as *V*_max_/*K*_m_) (Brouwer et al. 1987) and the highest catalytic efficiency (Kouzuki et al. 1999). Based on the method reported by Evans and Andersen (2000) the sensitivity coefficients (SCs) for the model parameters were calculated as follows:

4 $$\mathrm{SC}=(C{^{\prime}}-C)/(P{^{\prime}}-P)\times P/C,$$

where *C* indicates the initial value of the model output, *C*′ indicates the modified value of the model output resulting from an increase in the parameter value. *P* indicates the initial parameter value and *P′* indicates the modified parameter value after a 5% increase of its value, keeping all other parameters at their original value.

## Results

### In vitro kinetic data

Data from the four studies reporting values for the kinetic parameters *V*_max_ and *K*_m_ for E_2_17βG transport by hepatocytes are listed in Table 3. For both the *K*_m_ and *V*_max_ the maximum fold difference between the values from the different data sets was not higher than 11. The resulting catalytic efficiencies (CE) vary less than 3.1-fold.

**Table 3:** In vitro kinetic parameter values for active hepatic transport of [^3^H]-E_2_17βG obtained from literature

Rat hepatocyte system	*K*_m_ (µM)	*V*_max_ (pmol/min/mg protein)	Catalytic efficiency (CE) (*V*_max_/*K*_m_)	References
1	4.54 ± 2.5	149 ± 9.5	32.8	Brouwer et al. (1987)
2	6.5 ± 1.6	470 ± 120	72.3	Ishizuka et al. (1998)
3	12.9 ± 1.3	1300 ± 100	100.7	Kouzuki et al. (1999)
4	45.5 ± 11.8	1620 ± 210	35.6	Brock and Vore (1984)

### Predicted versus observed time-dependent blood levels of E_2_17βG

**Fig. 3:**
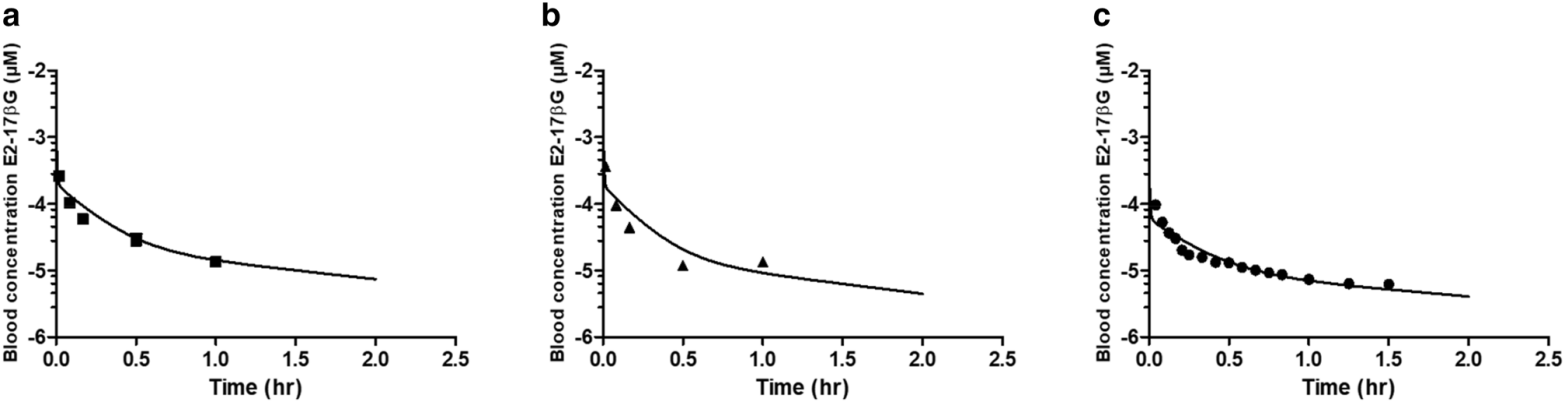
Predicted and observed blood concentrations (corrected from reported plasma concentrations) of E_2_17βG in rats upon intravenous administration. Symbols represent rat in vivo data obtained at a dose of (**a**) 81 ng/kg bw (squares) (Morikawa et al. 2000), (**b**) 81 ng/kg bw (triangles) (Gotoh et al. 2002) and (**c**) 23 ng/kg bw (dots) (Slikker et al. 1983). Data represent the mean (and the SD where available). Predictions (lines) are based on the *V*_max_ and *K*_m_ values for hepatocyte transport of E_2_17βG from rat hepatocyte system 1 obtained from literature and presented in Table 3. The predictions for all four *V*_max_ and *K*_m_ data sets are presented in supplementary material A figure S3. The details of the in vivo data sets are presented in Table 2

Using the *V*_max_ and *K*_m_ values presented in Table 3, PBK model-based predictions for E_2_17βG levels in blood were calculated optimising the scaling factor to obtain the best fit between predicted and actually reported experimental data for the three available in vivo data sets (Table 2). To this end an iterative process varying the scaling factor within the range of 5–500 mg protein/g liver was performed to find the best fit. For each in vivo data set four optimisations were carried out, one for each of the data sets for *V*_max_ and *K*_m_ derived from rat hepatocytes (Table 3). Since the four fitted predictions thus obtained for each of the three experimentally observed time-dependent blood concentration of intravenously administered E_2_17βG in rats were similar, only the predictions for one out of the four data sets are displayed in Fig. 3 (all twelve predictions can be found in supplementary material A fig. S3). The scaling factors in mg protein/g liver for conversion of in vitro hepatocyte protein levels to in vivo liver protein levels optimised to obtain the predicted curves are presented in Table 4.

**Table 4:** Fitted values for the scaling factor and the resulting average scaling factor (mg protein/g liver)

	Morikawa et al. (2000)	Gotoh et al. (2002)	Slikker et al. (1983)	Morikawa et al. (2000)	Gotoh et al. (2002)	Takikawa et al. (1996)
Rat hepatocyte system	Blood concentration			Cumulative biliary excretion		
	Scaling factor (mg protein/g liver)			Scaling factor (mg protein/g liver)		
1^a^	80	200	35	200	65	400
2^b^	40	90	15	150	30	200
3^c^	30	65	11	120	22	200
4^d^	80	180	30	300	60	400
Average scaling factor ± SEM (mg protein/g liver)	129 ± 24					

Scaling factors were obtained by fitting the in vivo reported data on blood and cumulative biliary excretion levels with the PBK-model predictions based on the in vitro kinetic input

^a^1: *K*_m_ = 4.54 µM, *V*_max_ = 149 pmol/min/mg protein (Brouwer et al. 1987)

^b^2: *K*_m_ = 6.5 µM, *V*_max_ = 470 pmol/min/mg protein (Ishizuka et al. 1998)

^c^3: *K*_m_ = 12.9 µM, *V*_max_ = 1300 pmol/min/mg/protein (Kouzuki et al. 1999)

^d^4: *K*_m_ = 45.5 µM, *V*_max_ = 1620 pmol/min/mg protein (Brock and Vore 1984)

The data thus obtained reveal that with all kinetic hepatocyte data adequate fits can be obtained with scaling factors that differ only to a limited extent for the four kinetic hepatocyte data sets. The scaling factors for the fits of the 3 different in vivo data sets for blood levels of E_2_17βG varied here amounting to at most a sixfold difference when comparing results *within* a hepatocyte data set.

### Predicted versus observed time-dependent cumulative biliary excretion of E_2_17βG

**Fig. 4:**
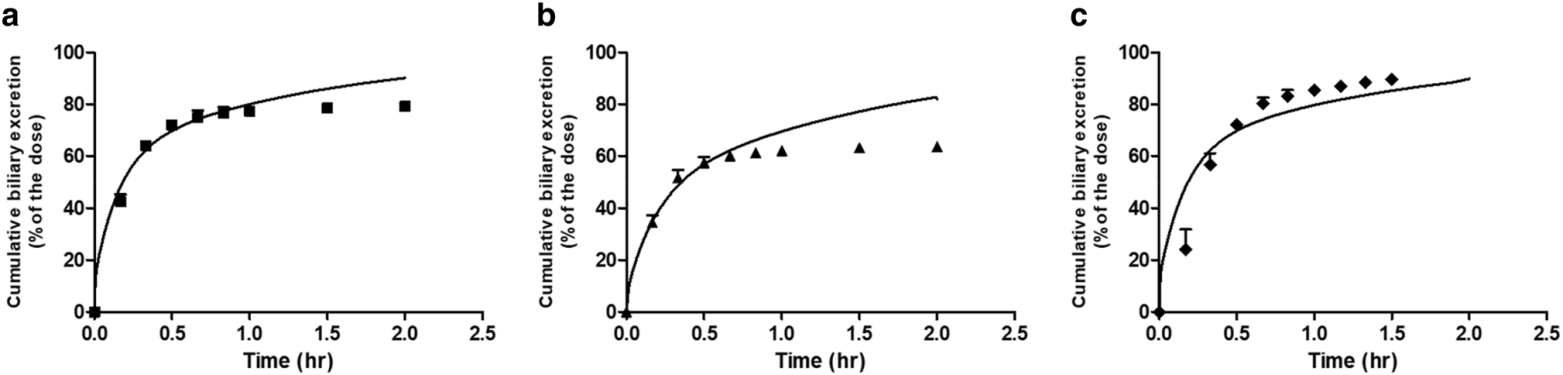
Predicted and observed cumulative biliary excretion of E_2_17βG in rats upon intravenous administration expressed as percentage of the dose (%). Symbols represent rat in vivo data obtained at a dose of (**a**) 81 ng/kg bw (squares) (Morikawa et al. 2000), (**b**) 81 ng/kg bw (triangles) (Gotoh et al. 2002) and (**c**) 48 ng/kg bw (diamonds) (Takikawa et al. 1996). Data represent the mean (and the SD where available). Predictions (lines) are based on the *V*_max_ and *K*_m_ values for hepatocyte transport of E_2_17βG from rat hepatocyte system 1 obtained from literature and presented in Table 3. The predictions for all four *V*_max_ and *K*_m_ data sets are presented in supplementary material A figure S4. The details of the in vivo data sets are presented in Table 2

Next to the time-dependent blood concentration, the cumulative biliary excretion was predicted using the same approach (Fig. 4). Since also for these three data sets on the time-dependent biliary excretion the predictions obtained using the four *V*_max_ and *K*_m_ data sets were similar, only the predictions for one out of the four data sets are displayed in Fig. 4. All twelve predictions can be found in supplementary material A fig. S4. The values for the scaling factor in mg protein/g liver for conversion of in vitro hepatocyte protein levels to in vivo liver protein levels optimised to obtain the predicted curves are presented in Table 4.

The data obtained reveal that also for prediction of the cumulative biliary excretion all kinetic hepatocyte data can provide adequate fits with scaling factors that differ only to a limited extent for the four kinetic hepatocyte data sets. The scaling factors for the fits of the 3 different in vivo data sets for blood E_2_17βG varied somewhat more compared with the scaling factors obtained from the time-dependent blood concentration amounting from a sixfold to a ninefold difference when comparing results *within* a hepatocyte data set. Taking all 24 scaling factors together resulted in an average scaling factor of 129 ± 24 mg protein/g liver (mean ± SEM).

### Evaluation of the scaling factor

**Fig. 5:**
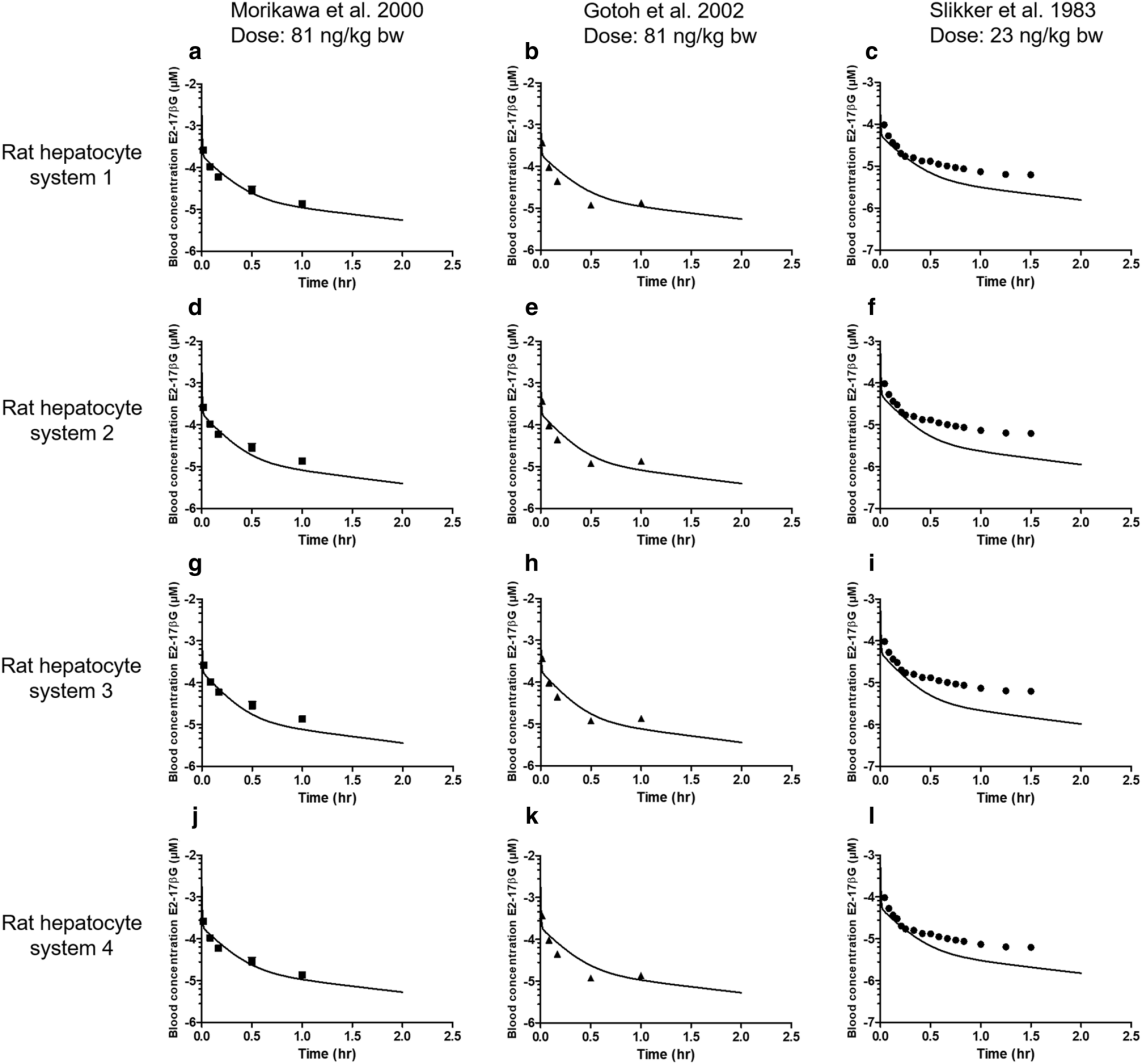
Predictions of the blood concentration in time of E_2_17βG in rats upon intravenous administration using the average value for the scaling factor (129 mg protein/g liver) compared with the observed blood concentrations. Symbols represent rat in vivo kinetic data obtained at a dose of (**a**, **d**, **g**, **j**) 81 ng/kg bw (squares) (Morikawa et al. 2000), (**b**, **e**, **h**, **k**) 81 ng/kg bw (triangles) (Gotoh et al. 2002) and (**c**, **f**, **i**, **l**) 23 ng/kg bw (dots) (Slikker et al. 1983). Data represent the mean (and the SD where available). Predictions (lines) are based on the *V*_max_ and *K*_m_ values for hepatocyte transport of E_2_17βG obtained from literature and presented in Table 3

**Fig. 6:**
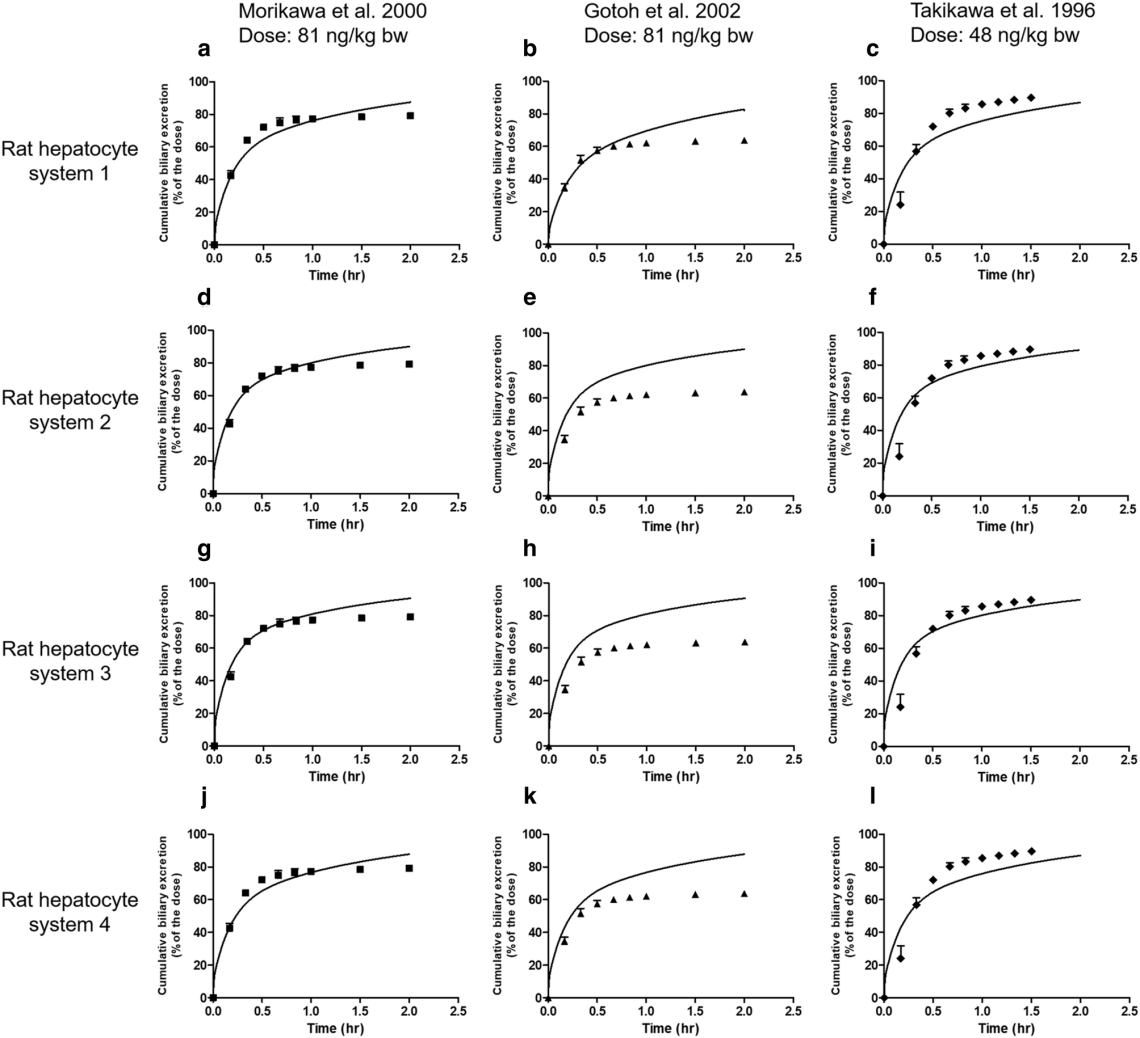
Predictions of the cumulative biliary excretion in time of E_2_17βG in rats upon intravenous administration expressed as percentage of the dose (%) using the average value for the scaling factor (129 mg protein/g liver) compared with the observed cumulative biliary excretion. Symbols represent rat in vivo kinetic data obtained at a dose of (**a**, **d**, **g**, **j**) 81 ng/kg bw (squares) (Morikawa et al. 2000), (**b**, **e**, h, **k**) 81 ng/kg bw (triangles) (Gotoh et al. 2002) and (**c**, **f**, **i**, **l**) 48 ng/kg bw (diamonds) (Takikawa et al. 1996). Data represent the mean (and the SD where available). Predictions (lines) are based on the *V*_max_ and *K*_m_ values for hepatocyte transport of E_2_17βG obtained from literature and presented in Table 3

To further evaluate the scaling factor, the in vivo kinetic data on time-dependent E_2_17βG blood concentration and cumulative biliary excretion were predicted using this average value and compared to the experimental data in Figs. 5, 6. The results obtained reveal that with this average scaling factor all experimental data for blood E_2_17βG levels were on average predicted with a less than 1.8-fold deviation, while for the data for the cumulative biliary excretion of E_2_17βG the averaged deviation was less than 1.4-fold. The largest deviations were observed for the in vivo data on blood E_2_17βG levels of Slikker et al. (1983) where the model somewhat overpredicted the clearance of E_2_17βG from the blood (Fig. 5c, f, i, l) ranging from a 4 to 12- fold difference and for the in vivo data on cumulative biliary excretion reported by Gotoh et al. (2002) that were too, somewhat overpredicted with a maximum of sixfold deviation (Fig. 6b, e, h, k).

### Sensitivity analysis

**Fig. 7:**
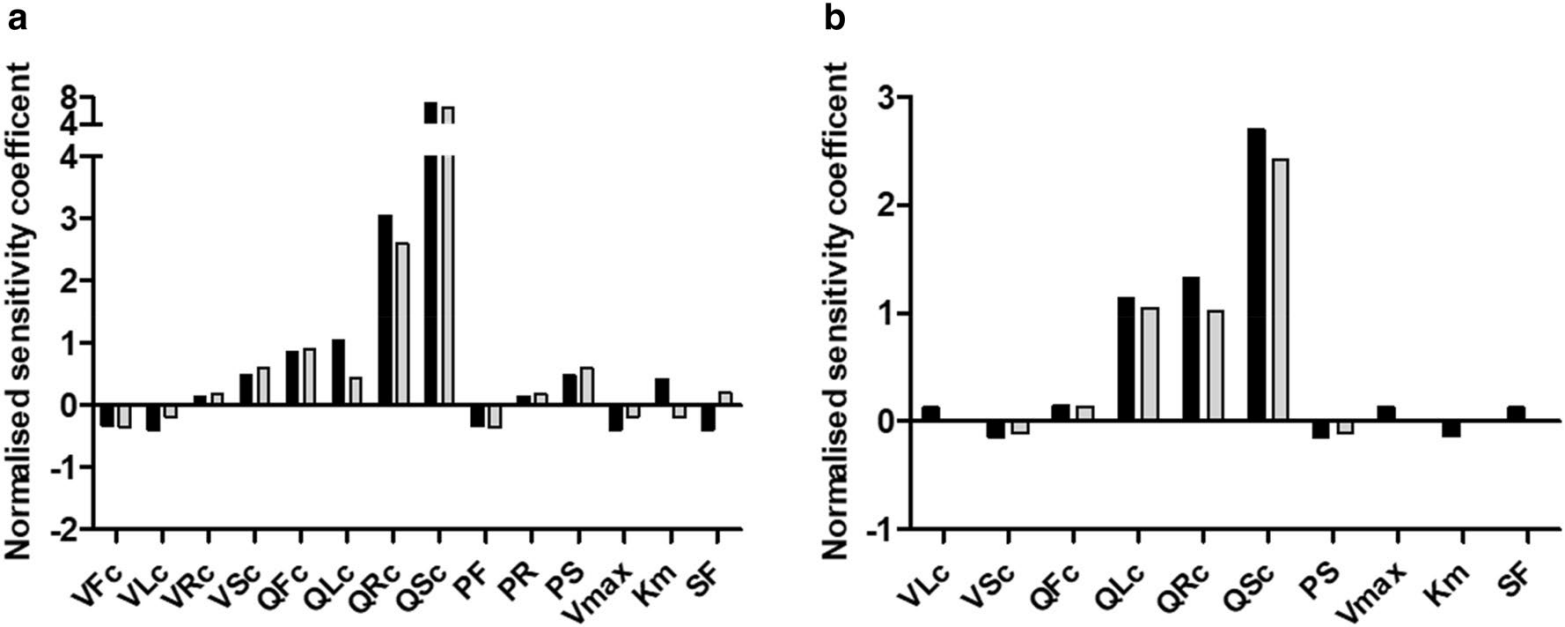
Sensitivity coefficients of the PBK-model parameters for prediction of **a** the E_2_17βG blood concentration and **b** the cumulative active biliary excretion of E_2_17βG at an intravenous dose of 81 ng/kw bw, *t* = 0.5 h with the *V*_max_ and *K*_m_ that result in the lowest catalytic efficiency (CE) (black bars) (Brouwer et al. 1987) and the *V*_max_ and *K*_m_ that result in the highest catalytic efficiency (grey bars) (Kouzuki et al. 1999) (see Table 3). VFc = volume fat tissue, VLc = volume liver tissue, VRc = volume of rapidly perfused tissues, VSc = volume of slowly perfused tissue, QFc = blood flow to fat, QLc = blood flow to liver, QRc = blood flow to rapidly perfused tissue, QSc = blood flow to slowly perfused tissue, PF = partition coefficient of fat, PR = partition coefficient of rapidly perfused tissue, PS = partition coefficient of slowly perfused tissue, *V*_max_ = maximum rate of E_2_17βG transport in hepatocytes, *K*_m_ = Michaelis–Menten constant of E_2_17βG transport in hepatocytes, SF = scaling factor

To further evaluate the influence of the scaling factor on the model predictions and also elucidate which PBK-model parameters influence the predictions most a sensitivity analysis was performed. The predicted blood concentration and cumulative biliary excretion levels of E_2_17βG at a time point of 0.5 h were used as the basis for this analysis. Only parameters with a normalized sensitivity coefficient > 0.1 are presented. The results reveal that for prediction of both the blood E_2_17βG concentration (Fig. 7a) and the cumulative biliary excretion (Fig. 7b) the blood flow to slowly perfused tissue (QSc) and the blood flow to rapidly perfused tissue (QRc) are most influential parameters while for prediction of the cumulative biliary excretion also the blood flow to the liver (QLc) is influential. At different catalytic efficiencies (CE) for the hepatocyte transport the normalized sensitivity coefficients are somewhat different, but the parameters that have the largest influence remain the same. The influence of the scaling factor on both model outcomes shows not to be substantial with a sensitivity coefficient at the highest and lowest CE of 0.2 and -0.4 for the blood concentration and 0.04 and 0.13 for the cumulative biliary excretion.

## Discussion

The aim of the present study was to provide a proof of principle for including biliary excretion into a generic rat PBK model with a major focus on determining the in vitro to in vivo scaling factor to be used when defining the kinetics of biliary transport using primary rat hepatocytes. A scaling factor is required to convert a kinetic value obtained in the in vitro cellular model, to a value that reflects the same biological function for the relevant whole organ. In this study, the scaling factor was needed to translate the in vitro *V*_max_ value for E_2_17βG transport in hepatocytes expressed in pmol/min/mg protein to an in vivo rate for E_2_17βG transport by the liver expressed in µmol/h. The scaling factor expressed in mg protein/g liver enabled use of the in vitro hepatocyte *V*_max_ value in the PBK model to enable prediction of in vivo blood and cumulative biliary excretion levels of intravenously administered E_2_17βG. We used E_2_17βG as a model substance because the substance is excreted as such, with its active excretion into bile via the activity of the Oatp transporters in the sinusoidal membrane of hepatocytes being the rate-limiting step for its elimination (Varma et al. 2012). Using the obtained value for the scaling factor, 129 mg protein/g liver, resulted in PBK model-based predictions of the blood and cumulative biliary excretion levels of E_2_17βG that were on average within a 1.8-fold deviation from reported in vivo data, thus showing the value of the use of primary hepatocytes as an in vitro system to determine kinetic parameters for describing biliary excretion.

It is of importance to note that main deviations between predicted and observed in vivo data could be related to especially differences between reported experimental data at the same dose level. Even though the in vivo time-dependent blood concentration and the corresponding cumulative biliary excretion reported by Gotoh et al. (2002) and Morikawa et al. (2000) were obtained from the same laboratory, the outcomes were different resulting in different values for the individual scaling factors obtained when fitting the data. This difference between the experimental data sets might be explained by the two year time-lap between the two studies and the fact that animal experiments are depending on an array of guideline protocols and conditions that might have been slightly changed over the two years influencing the (expected) outcome (Council 2011). This deviation in reported in vivo data becomes even more apparent when looking at the in vivo data from Slikker et al. (1983) where at one third of the dose level the data for E_2_17βG blood levels were overlapping with the data of the two studies using a threefold higher dose level. This discrepancy could be related to the sex of the rats used (female not male) in the Slikker et al. (1983) study. However, Gotoh et al. (2002) reported in vivo data on blood levels of E_2_17βG in female Sprague–Dawley rats, and showed that these levels were comparable to the levels found in the male Sprague–Dawley rats. Other factors that could play a role in the differences in in vivo data are the vehicle used for the IV administration, which was distilled water: polyethylene glycol: ethanol (10:4:1 v/v) in the studies reported by Gotoh et al. (2002) and Morikawa et al. (2000) but saline: propylene glycol: ethanol (10:4:1 v/v) in the study of Slikker et al. (1983). Use of a different vehicle may influence the bioavailability, which may have been higher in the Slikker et al. (1983) study. Furthermore, the type and rate of IV administration, which for one study was an IV infusion (Takikawa et al. 1996) while for the other three studies a bolus IV administration was utilized.

In the present study the apparent deviation shown in the data reported by Slikker et al. (1983) was corrected for before using the data to define the scaling factor. Without this correction scaling factors obtained for the Slikker et al. (1983) data set appeared to amount to a maximum 10 mg protein/g liver, representing values that are not substantially different from the ones reported in Table 4. This observation corroborates the results of the sensitivity analysis that revealed that the scaling factor was not an influential parameter for the PBK model-based predictions.

Until now, not much has been published about the scaling factor expressed in mg protein/g liver to convert in vitro hepatocyte protein levels to in vivo liver data in rats. A study by Sohlenius-Sternbeck (2006) reported a protein concentration in rat liver homogenate of 112 mg/g liver, but did not define how that value relates to primary hepatocytes, the model system of the present study. However, to further support the scaling factor now obtained the following theoretical approach could be applied to approximate the scaling factor by the use of a hepatocellularity number. There are a few studies that determined and reported this number ranging from 117 to 135 × 10^6^ cells (hepatocytes)/g liver (Bayliss et al. 1999; Houston 1994, Sohlenius-Sternbeck 2006). Sohlenius-Sternbeck (2006) also reported on the protein concentration in a hepatocyte suspension (0.985 mg/10^6^ cells). Together with the weight of the liver (Table 1) these numbers would result in a scaling factor ranging from of 115 to 132 mg protein/g liver. Our modelled and averaged scaling factor based on 24 fitted predictions using in vitro input data from rat hepatocytes on active uptake of E_2_17βG and in vivo kinetic data is fully in line with this theoretical estimate.

The predictions made using the defined scaling factor revealed (1) that the value 129 mg protein/g liver is suitable to translate the in vitro *V*_max_ from hepatocytes to an in vivo *V*_max_ in liver obtaining predictions that are in line with the in vivo kinetic data for blood and cumulative biliary excretion levels of E_2_17βG and also (2) that hepatocytes provide an adequate in vitro model to obtain kinetic parameters *V*_max_ and *K*_m_ for active uptake of an Oatp substrate, E_2_17βG in our study. Given the uncertainty in scaling factors that would be required when using transfected cell models, use of hepatocytes to describe biliary excretion may be preferred over the use of in vitro systems with an overexpression of individual transporters. This is also because E_2_17βG has affinity for more than one Oatp which will all be taken into account when using hepatocytes known to contain multiple Oatps (e.g. 1a1, 1a5, 361 1b2, 2b1) thus better mimicking the overall transport in the organ of interest (Hagenbuch and Meier 2003; Richert et al. 2006). Taking all together it is concluded that freshly isolated hepatocytes provide an adequate in vitro system to investigate uptake via active transport resulting in kinetic parameters that are able to include biliary excretion into PBK-models (Harris et al. 2004; Sahi et al. 2010). Use of this model also eliminates the need for the use of REFs and RAFs to scale expression and activity of transporters from in vitro systems to a full organ, diminishing uncertainties.

With this study we demonstrated a proof of principle by introducing a novel testing strategy for including biliary excretion into a generic PBK model at the same time defining the scaling factor for the vitro system (primary rat hepatocytes) used to obtain adequate in vitro to in vivo translation. We confirmed based on the predictions using the averaged scaling factor that primary rat hepatocytes can be the gold standard for studies on biliary transport in addition to their use as the golden standard for many in vitro (human) hepatic endpoints (e.g. hepatic metabolism, hepatoxicity, induction/inhibition of cytochrome P450s) (Guguen-Guillouzo and Guillouzo 2010; Zeilinger et al. 2016). However, known downfalls of the use of primary hepatocytes are the inter individual differences and their non-high through put character. To what extent such bottlenecks would affect their use as a model for biliary transport remains to be elucidated. This also holds for their use to mimic biliary transport of other substrates for Oatps and substrates for other hepatic transporters (Oat2 and Oct1) involved in biliary excretion. To this end similar studies with other substrates for Oatps such as statins and ACE inhibitors (Izumi et al. 2018; Watanabe et al. 2010) or for other hepatic transporters such as antineoplastic drugs, antivirals. antidiuretics and some alkaloids (Burckhardt 2012; Lozano et al. 2013; Marada et al. 2015) may be of use. Nevertheless, the proof of principle described in the present paper provides a first important step towards including biliary excretion in PBK-models for reverse dosimetry based QIVIVE and alternative testing strategies.

## Electronic supplementary material

Below is the link to the electronic supplementary material.

Supplementary file1 (DOCX 302 kb)

Supplementary file2 (DOCX 17 kb)
