# Supplementary material for: Novel testing strategy for prediction of rat biliary excretion of intravenously administered estradiol-17β glucuronide
Source: Arch Toxicol. 2020 Nov 7;95(1):91–102. doi: 10.1007/s00204-020-02908-x (PMC7811516; doi:10.1007/s00204-020-02908-x)
Supplement: Supplementary file 1 — Supplementary file1 (DOCX 302 kb) [file 204_2020_2908_MOESM1_ESM.docx]

Novel testing strategy for prediction of rat biliary excretion of intravenously administered estradiol-17β glucuronide

Archives of Toxicology

Annelies Noorlander^1^, Eric Fabian^2^, Bennard van Ravenzwaay^2^, Ivonne M.C.M. Rietjens^1^

1. Division of Toxicology, Wageningen University and Research, Wageningen, The Netherlands
2. Experimental Toxicology and Ecology, BASF SE, Ludwigshafen, Germany.

Corresponding author: annelies.noorlander@wur.nl

**Supplementary material A**


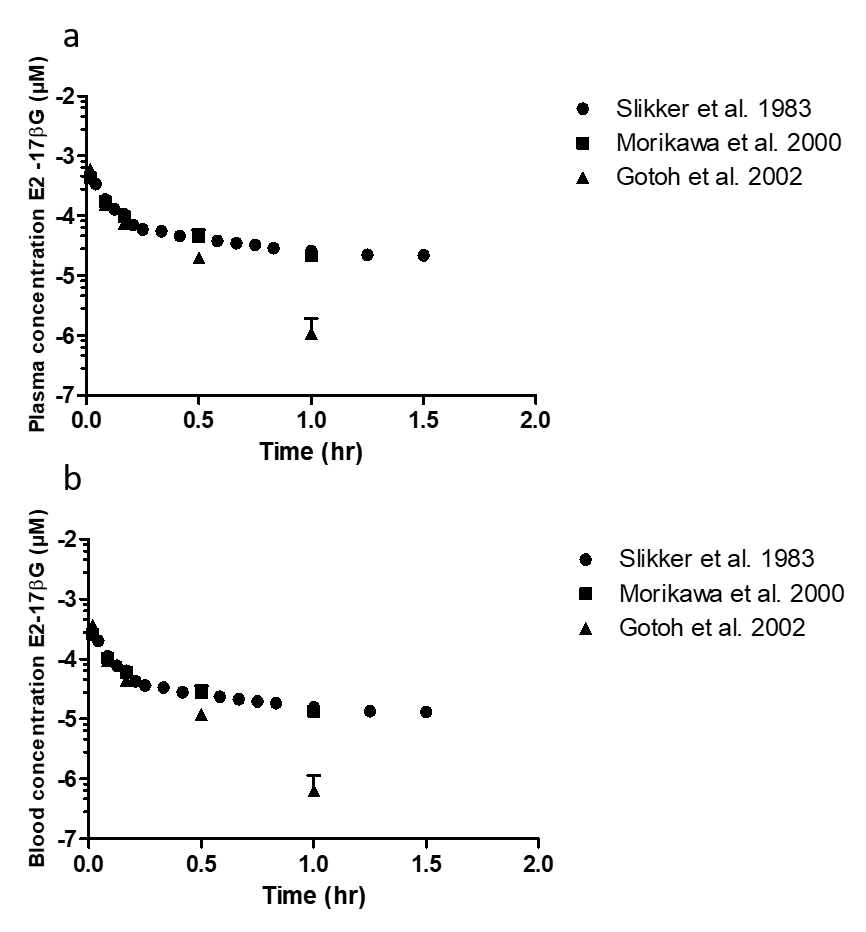


**Fig. S1** Original plasma concentration data (a) of E_2_17βG obtained from three different in vivo data sets at dose levels of 23 ng/kg bw (Slikker et al. 1983) (circles) and 81 ng/kg bw (Gotoh et al. 2002; Morikawa et al. 2000) (triangles and squares, respectively). Calculated whole blood concentration data (b) with the formula C_blood_ = C_plasma_ x (1-Hct) using 40% as the haematocrit in rat.


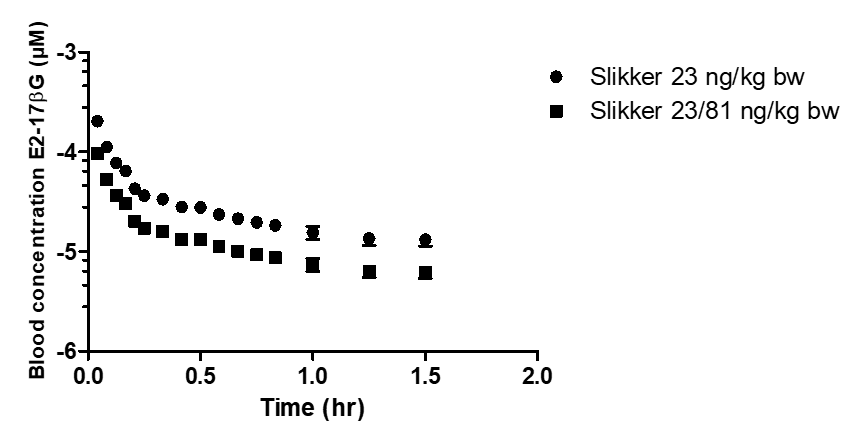


**Fig. S2** The in vivo data of Slikker et al. (1983) where the plasma to blood converted data (circles) have been modified 23/81 (squares).


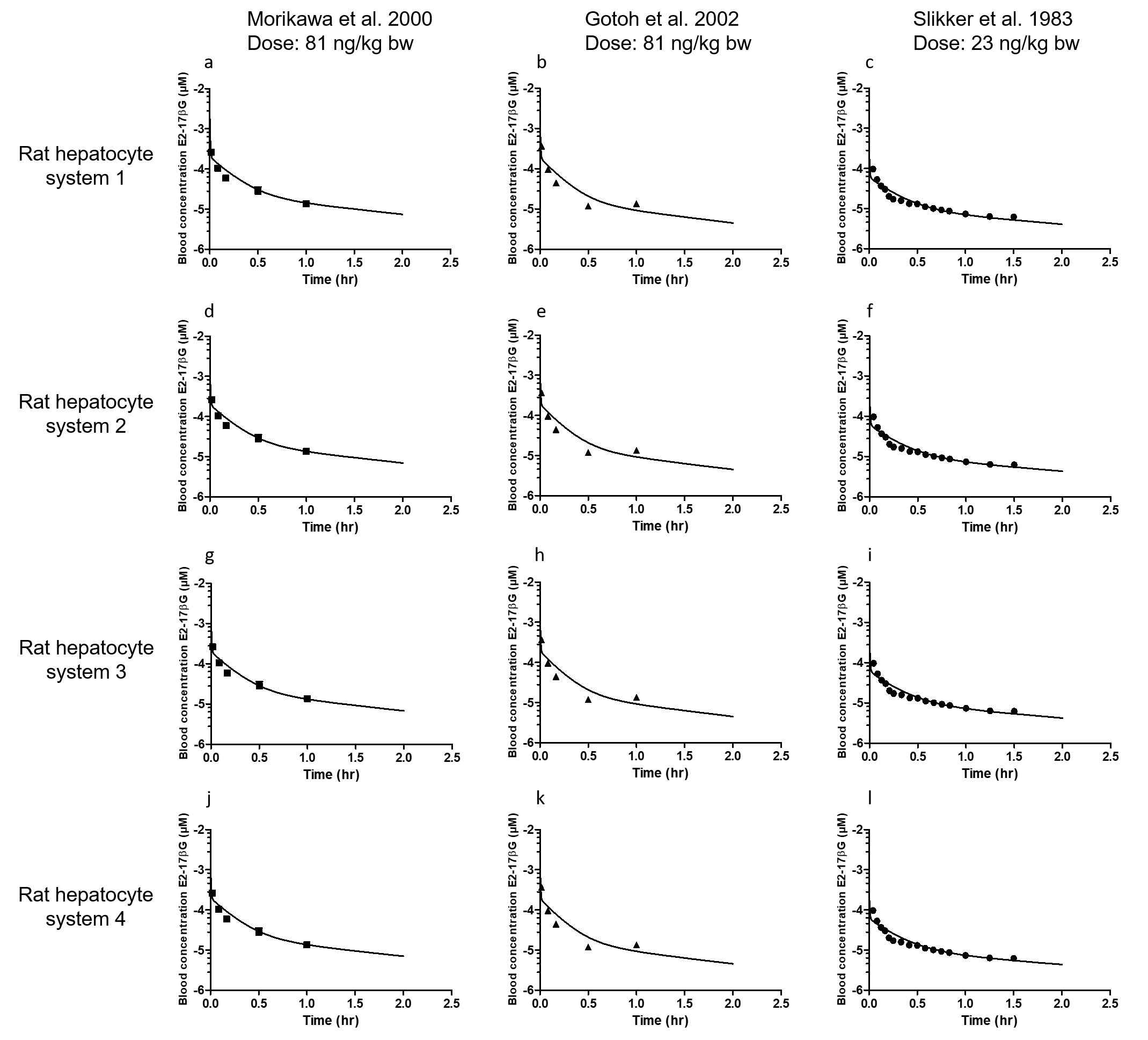


**Fig. S3** Fitted predictions and observed blood concentrations (corrected from reported plasma concentrations) of E_2_17βG in rats upon intravenous administration. Symbols represent rat in vivo data obtained at a dose of (a, d, g, j) 81 ng/kg bw (squares) (Morikawa et al. 2000), (b, e, h, k) 81 ng/kg bw (triangles) (Gotoh et al. 2002) and (c, f, i, l) 23 ng/kg bw (dots) (Slikker et al. 1983). Data represent the mean and the SD where available. Predictions (lines) are based on the Vmax and Km values for hepatocyte transport of E_2_17βG obtained from literature and presented in table 3


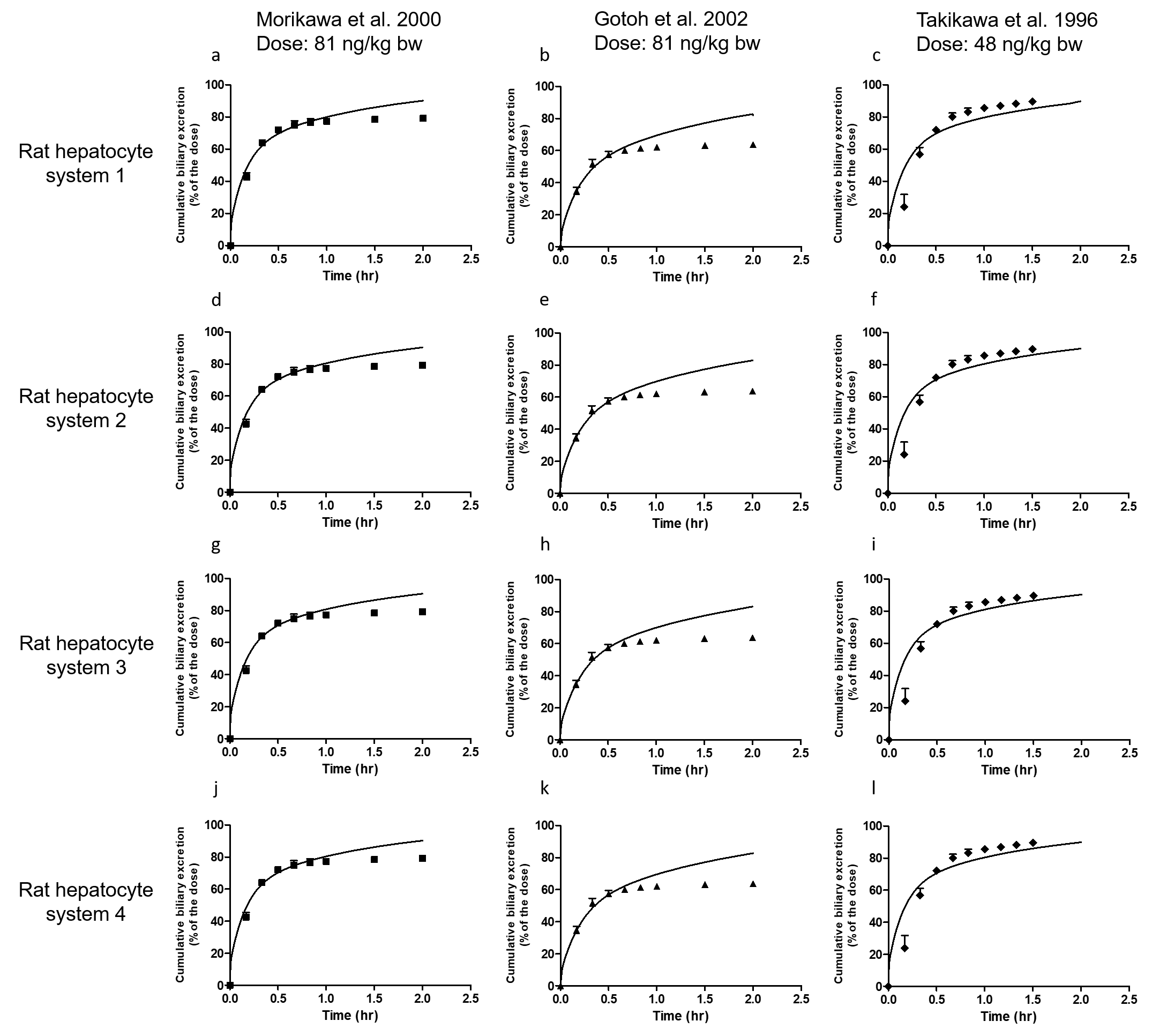


**Fig. S4** Fitted predictions and observed cumulative biliary excretion of E_2_17βG in rats upon intravenous administration. Symbols represent rat in vivo data obtained at a dose of (a, d, g, j) 81 ng/kg bw (squares) (Morikawa et al. 2000), (b, e, h, k) 81 ng/kg bw (triangles) (Gotoh et al. 2002) and (c, f, i, l) 48 ng/kg bw (diamonds) (Takikawa et al. 1996). Data represent the mean and the SD where available. Predictions (lines) are based on the Vmax and Km values for hepatocyte transport of E_2_17βG obtained from literature and presented in table 3

Gotoh Y, Kato Y, Stieger B, Meier PJ, Sugiyama Y (2002) Gender difference in the Oatp1-mediated tubular reabsorption of estradiol 17beta-D-glucuronide in rats. Am J Physiol Endocrinol Metab 282(6):E1245-54 doi:10.1152/ajpendo.00363.2001

Morikawa A, Goto Y, Suzuki H, Hirohashi T, Sugiyama Y (2000) Biliary excretion of 17beta-estradiol 17beta-D-glucuronide is predominantly mediated by cMOAT/MRP2. Pharm Res 17(5):546-52

Slikker W, Jr., Vore M, Bailey JR, Meyers M, Montgomery C (1983) Hepatotoxic effects of estradiol-17 beta-D-glucuronide in the rat and monkey. J Pharmacol Exp Ther 225(1):138-43

Takikawa H, Yamazaki R, Sano N, Yamanaka M (1996) Biliary excretion of estradiol-17 beta-glucuronide in the rat. Hepatology 23(3):607-13 doi:10.1053/jhep.1996.v23.pm0008617443
