# Supplementary material for: Novel testing strategy for prediction of rat biliary excretion of intravenously administered estradiol-17β glucuronide
Source: Arch Toxicol. 2020 Nov 7;95(1):91–102. doi: 10.1007/s00204-020-02908-x (PMC7811516; doi:10.1007/s00204-020-02908-x)
Supplement: Supplementary file 2 — Supplementary file2 (DOCX 17 kb) [file 204_2020_2908_MOESM2_ESM.docx]

Novel testing strategy for prediction of rat biliary excretion of intravenously administered estradiol-17β glucuronide

Archives of Toxicology

Annelies Noorlander^1^, Eric Fabian^2^, Bennard van Ravenzwaay^2^, Ivonne M.C.M. Rietjens^1^

1. Division of Toxicology, Wageningen University and Research, Wageningen, The Netherlands
2. Experimental Toxicology and Ecology, BASF SE, Ludwigshafen, Germany.

Corresponding author: annelies.noorlander@wur.nl

**Supplementary material B. The Berkeley Madonna model code used.**

; Date: January 2019

; Purpose: PBK Model of estradiol-17B-glucuronide (E217G), built with in vitro (from literature) and in silico derived parameter values

; Species: Rat

; Compiled by: Annelies Noorlander

; Organisation: Wageningen University

;=====================================================================

;Physiological parameters

;=====================================================================

; tissue volumes

BW = 0.250 {Kg} ; body weight rat (variable, dependent on study)

VFc = 0.07 ; fraction of fat tissue reference: Brown et al. Table 21 (1997)

VLc = 0.034 ; fraction of liver tissue reference: Brown et al. Table 21 (1997)

VBc = 0.074 ; fraction of blood reference: Brown et al. Table 21 (1997)

VRc = 0.098 ; fraction of richly perfused tissue reference: Brown et al. Table 21 (1997)

VSc = 0.724 ; fraction of slowly perfused tissue reference: Brown et al. Table 21 (1997)

VF = VFc*BW {L or Kg} ; volume of fat tissue (calculated)

VL = VLc*BW {L or Kg} ; volume of liver tissue (calculated)

VB = VBc*BW {L or Kg} ; volume of blood (calculated)

VR = VRc*BW {L or Kg} ; volume of richly perfused tissue (calculated)

VS = VSc*BW {L or Kg} ; volume of slowly perfused tissue (calculated)

;--------------------------------------------------------------------------------------------------------------------

;blood flow rates

QC = 15*(BW)^0.74 {L/hr} ; cardiac output reference: Brown et al. p.453 (1997)

QFc = 0.07 ; fraction of blood flow to fat reference: Brown et al. Table 25 (1997)

QLc = 0.174 ; fraction of blood flow to liver reference: Brown et al. Table 25 (1997)

QRc = 0.234 ; fraction of blood flow to richly perfused tissue reference: Brown et al. Table 25 (1997)

QSc = 0.522 ; fraction of blood flow to slowly perfused tissue reference: Brown et al. Table 25 (1997).

QF = QFc*QC {L/hr} ; blood flow to fat tissue (calculated)

QL = QLc*QC {L/hr} ; blood flow to liver tissue (calculated)

QS = QSc*QC {L/hr} ; blood flow to slowly perfused tissue (calculated)

QR = QRc*QC {L/hr} ; blood flow to richly perfused tissue (calculated)

;=====================================================================

;Physicochemical parameters

;=====================================================================

;partition coefficients --> logP E217G from ALOGPS: 2.05

PF = 19.87452 ; fat/blood partition coefficient calculated using QPPR of: (DeJongh, 1997)

PL = 1.332858 ; liver/blood partition coefficient calculated using QPPR of: (DeJongh, 1997)

PR = 1.332858 ; richly perfused tissue/blood partition coefficient calculated using QPPR of: (DeJongh, 1997)

PS = 0.600644 ; richly perfused tissue/blood partition coefficient calculated using QPPR of: (DeJongh, 1997)

;=====================================================================

;Kinetic parameters

;=====================================================================

;Transport from needle to blood

kn = 1000000 ; injection {/hr}

;--------------------------------------------------------------------------------------------------------------------

;Biliary excretion from liver

;based on uptake and elimination of E217G via active transporters in rat hepatocytes (Oatps)

VmaxE217Gc= 149 {pmol/min/mg} ; data derived from Brouwer et al. 1987

SF = 129 {mg/g liver} ;Averaged scaling factor

VMaxE217G = (VMaxE217Gc/1000000)*SF*60*VL*1000 {umol/hr}

;E217G uptake and elimination, affinity constants for uptake transporters in rat hepatocytes (Oatps) (umol/L)

KmE217G = 4.54 ; data derived from Brouwer et al. 1987

;=====================================================================

;Run settings

;=====================================================================

;Molecular weight E217G

MW = 448.512

;--------------------------------------------------------------------------------------------------------------------

;IV dose

IVDOSEmg = 0.000081 {mg/kg bw} ; IVDOSEmg = given IV dose in mg/kg bw

IVDOSEumol2 = IVDOSEmg*1E-3/MW*1E6 {umol/kg bw} ; IVDOSEumol2 = given IV dose recalculated to umol/kg bw

IVDOSEumol=IVDOSEumol2*BW; ; IVDOSEumol = umol given IV

;time

Starttime = 0 ; in hr

Stoptime = 24 ; in hr

;=====================================================================

;Model calculations

;=====================================================================

; Model E217G

;needle compartment

;ANe = amount in needle, umol

;ANe' = Change in amount of E217G in time in the needle, umol/hr

ANe' = -kn*ANe

Init ANe = IVDOSEumol

;--------------------------------------------------------------------------------------------------------------------

;liver compartment

;AL = Amount E217G in liver tissue, umol

;AL' = Change in amount of E217G in time, umol/hr

AL' = QL*(CB - CVL) - AME217G'

Init AL = 0

CL = AL/VL

CVL = CL/PL

;AME217G = amount E217G excreted from the liver by biliary excretion over time (umol/L)

AME217G' = VmaxE217G*CVL/(KmE217G + CVL)

init AME217G = 0

;--------------------------------------------------------------------------------------------------------------------

;fat compartment

;AF = Amount E217G in fat tissue (umol)

;AF' = Change in amount of E217G in time, umol/hr

AF' = QF*(CB-CVF)

Init AF = 0

CF = AF/VF

CVF = CF/PF

;--------------------------------------------------------------------------------------------------------------------

;tissue compartment richly perfused tissue

;AR = Amount E217G in richly perfused tissue (umol)

;AR' = Change in amount of E217G in time, umol/hr

AR' = QR*(CB-CVR)

Init AR = 0

CR = AR/VR

CVR = CR/PR

;--------------------------------------------------------------------------------------------------------------------

;tissue compartment slowly perfused tissue

;AS = Amount E217G in slowly perfused tissue (umol)

;AS' = Change in amount of E217G in time, umol/hr

AS' = QS*(CB-CVS)

Init AS = 0

CS = AS/VS

CVS = CS/PS

;--------------------------------------------------------------------------------------------------------------------

; blood compartment

;AB = Amount E217G in blood (umol)

;AB' = Change in amount of E217G in time, umol/hr

AB' = (kn*ANe + QF*CVF + QL*CVL + QS*CVS + QR*CVR - QC*CB)

Init AB = 0

CB = AB/VB

;=====================================================================

;Mass balance calculations

;=====================================================================

Total = IVDOSEumol

Calculated = ANe + AL + AME217G + AF + AS + AR + AB

ERROR=((Total-Calculated)/Total+1E-30)*100

MASSBBAL=Total-Calculated + 1
